# Xanthogranulomatous Prostatitis Mimicking Prostate Carcinoma in A 78‐Year‐Old Male

**DOI:** 10.1002/ccr3.71687

**Published:** 2025-12-14

**Authors:** Mohammed Salah E. Khalifa Salem, Eesaa Docrat, Lesego Molwelang, Pule David Molebatsi, Alain Mwamba Mukendi

**Affiliations:** ^1^ Division of Urology, Department of Surgery, Klerksdorp Hospital University of the Witwatersrand Klerksdorp South Africa

**Keywords:** diagnostic dilemma, multiparametric MRI, prostate adenocarcinoma, prostatitis, xanthogranulomatous

## Abstract

Prostate cancer is typically the leading diagnosis in patients with elevated PSA and abnormal digital rectal exam findings. Clinicians should consider mimickers such as xanthogranulomatous prostatitis in the differential diagnosis, particularly in cases with discordant PSA levels and imaging characteristics. A multidisciplinary approach integrating clinical, radiological, and histopathological findings is essential to avoid misdiagnosis and ensure accurate management.

## 1 Introduction

Xanthogranulomatous prostatitis, a rare and intriguing histopathological entity, poses a significant diagnostic challenge due to its unsettling resemblance to prostate adenocarcinoma in clinical presentation, biochemical markers, and even histopathological features. This uncommon condition, more frequently observed in organs like the kidneys and gallbladder, can masquerade as prostate cancer, leading to diagnostic dilemmas [1].

In this case report, we present a compelling instance where xanthogranulomatous prostatitis mimicked prostate adenocarcinoma, highlighting the importance of a multidisciplinary approach involving urologists, histopathologists, and radiologists. Furthermore, this case underscores the crucial role of multiparametric magnetic resonance imaging in identifying and characterizing suspicious prostatic lesions, thereby supporting its utility in guiding biopsy decisions [1].

## 2 Case History and Examination

A 78‐year‐old Black African male from the Northwest province of South Africa, referred to the urology outpatient department at Klerksdorp Hospital in May 2023 for evaluation of a markedly elevated prostate‐specific antigen (PSA) level exceeding 100 ng/mL. The patient had a background of well‐controlled hypertension and was an ex‐smoker with occasional alcohol use. He had no prior surgical history and only one previous hospital admission, which occurred in November 2022 for COVID‐19 pneumonia with concern for pulmonary embolism (PE).

During that admission, a contrast‐enhanced CT scan of the chest was done to assess for PE. Although no pulmonary embolism was detected, the scan incidentally noted sclerotic vertebral bony lesions with multilevel compression fractures, along with prostatomegaly. These findings raised concern for possible metastatic prostate cancer. When assessed in the urology clinic, the patient reported no lower urinary tract symptoms or systemic complaints. Digital rectal examination (DRE) revealed a very firm, nodular prostate consistent with a clinical T3 lesion. Neurological examination was unremarkable, and the patient was mobilizing independently with preserved strength and tone.

## 3 Diagnosis, Investigations and Treatment

**FIGURE 1:**
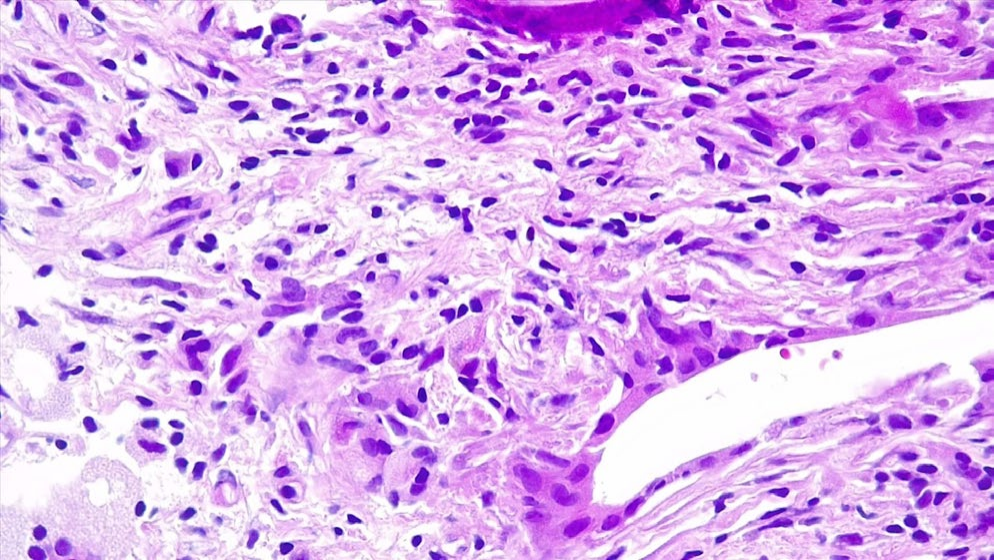
H&E images showing a diffuse histiocytic response comprising variable proportions of epithelioid histiocytes, foamy histiocytes and multinucleated giant cells. (original magnification ×400).

Given the elevated PSA, suspicious DRE, and imaging findings suggesting possible skeletal metastases, the patient was urgently scheduled for a prostate biopsy and was empirically commenced on androgen deprivation therapy (ADT) due to the high clinical suspicion for advanced prostate cancer and concern for potential spinal cord compression. The initial transrectal ultrasound‐guided biopsy, however, revealed histological features consistent with xanthogranulomatous prostatitis, without any evidence of malignancy, high‐grade prostatic intraepithelial neoplasia (HGPIN), or active inflammation. Due to concerns about the representativeness of the sample, a repeat biopsy was performed, which once again demonstrated xanthogranulomatous prostatitis with no malignant features (Figure 1).

A repeat CT scan showed no interval change in the vertebral lesions. Magnetic resonance imaging (MRI) of the prostate was done after the prostate biopsy due to it not being readily available in our low‐resource settings, with waiting times ranging from 3 to 6 months. MRI revealed an enlarged gland with a bulky transition zone but no focal mass or abnormal signal intensity to suggest malignancy (Video 1). A tuberculosis (TB) work‐up, including sputum and urine testing, was performed to rule out extrapulmonary TB as a possible cause of the vertebral lesions and prostatic inflammation; all results were negative.

**VIDEO 1:** Coronal view of a T2 weighted MRI pelvis demonstrating an enlarged prostate with a bulky transition zone. No distinct mass or area of abnormal tissue signal intensity noted. Video content can be viewed at https://onlinelibrary.wiley.com/doi/10.1002/ccr3.71687.

## 4 Outcome and Follow Up

The patient remained clinically stable throughout, with no neurological compromise or progression of symptoms. A repeat PSA test in October 2024 showed a marked decline to 0.44 ng/mL while on ADT. Given the persistently negative biopsies, no evidence of malignancy on imaging, and excellent clinical response, the decision was made to discontinue ADT. The patient is currently under PSA surveillance and remains asymptomatic and neurologically intact at follow‐up appointments. They were referred to orthopedic surgery for further assessment and management of spinal lesions. It had been brought to our attention that the patient had declined to consent for any vertebral biopsies on the basis that he had no spinal issues.

## 5 Discussion

Xanthogranulomatous prostatitis remains an uncommon entity and a notable mimicker of prostate adenocarcinoma [1, 2] The clinical presentation, laboratory results, and imaging features in this case strongly resembled those of advanced prostate adenocarcinoma. Ultimately, xanthogranulomatous prostatitis was confirmed as the definitive diagnosis based on histopathology evaluation of two consecutive biopsy specimens and lack of detection by MRI of suspicious lesions.

This prostatic condition is characterized by an abundance of histiocytes (foamy macrophages) admixed with numerous plasma cells and lymphocytes. The prominent histiocytic component can lead to diagnostic confusion, potentially mimicking adenocarcinoma of the prostate with a hypernephroid pattern [2].

Multiparametric MRI (mpMRI) can identify areas that may be missed by systematic transrectal ultrasound‐guided biopsy, providing valuable characterization of prostatic lesions. Features suggestive of xanthogranulomatous prostatitis on mpMRI include diffuse changes involving over 50% of the prostate gland, infiltration of periprostatic fat or extracapsular extension, and large areas of non‐enhancement corresponding to caseous abscesses. Nonetheless, distinguishing granulomatous prostatitis from adenocarcinoma remains a diagnostic challenge [1, 3]. In this case, however, the mpMRI of the prostate didn't show any suspicious lesion.

Histopathological examination plays a crucial role in accurately diagnosing prostatic lesions, particularly in the presence of elevated PSA levels and abnormal digital rectal examination. In cases where histopathological diagnosis is challenging, immunohistochemistry staining can be instrumental in distinguishing xanthogranulomatous prostatitis from carcinoma. Notably, xanthogranulomatous prostatitis typically shows negativity for broad‐spectrum cytokeratins and positivity for CD68, helping to confirm the diagnosis [1, 4, 5]. In this case, given findings suggestive of metastatic prostate adenocarcinoma, a repeat biopsy was performed after the initial biopsy revealed xanthogranulomatous prostatitis, and the subsequent biopsy results remained consistent.

Xanthogranulomatous prostatitis has been reported in the literature both in isolation and in conjunction with other conditions, such as prostatic abscess. However, in our case, ultrasound examination did not reveal any abscess. Additionally, a reported case of xanthogranulomatous prostatitis complicated by a prostato‐rectal fistula highlighted potential pathways for this complication, including spontaneous formation via abscess perforation into the rectum or as a consequence of transurethral resection of the prostate (TURP) [1, 6].

The management of xanthogranulomatous prostatitis typically involves conservative approaches, including alpha blockers and corticosteroids. In cases where symptoms are severe or conservative management is ineffective, surgical interventions such as transurethral resection of the prostate (TURP) or open prostatectomy may be indicated [1, 6]. Our patient did not require treatment for xanthogranulomatous prostatitis, as he was asymptomatic for lower urinary tract symptoms. However, due to initial concerns for metastatic prostate adenocarcinoma, androgen deprivation therapy was started but subsequently discontinued after malignancy was ruled out.

A multidisciplinary approach is crucial in accurately diagnosing complex conditions like xanthogranulomatous prostatitis. It ensures precise diagnosis and prevents unnecessary treatments through collaboration between clinicians, radiologists, and pathologists.1In this case scenario, a diagnostic dilemma arose from the discrepancy between clinical, laboratory, and initial imaging findings, which suggested malignancy, and the histopathological and multiparametric MRI results, which ultimately ruled out prostate adenocarcinoma. As the diagnosis of both conditions relies on histological examination, the histopathological report was pivotal in establishing xanthogranulomatous prostatitis as the definitive diagnosis, with mpMRI findings providing additional support.

## 6 Conclusion

This case illustrates the diagnostic challenge posed by xanthogranulomatous prostatitis, a rare benign condition that can closely mimic metastatic prostate cancer. It underscores the importance of histological confirmation, especially in cases with discordant clinical and radiological findings, and highlights the need to consider non‐malignant causes, even in the presence of significantly elevated PSA levels and suspicious imaging findings.

## Author Contributions

**Mohammed Salah E. Khalifa Salem:** writing – original draft. **Eesaa Docrat:** writing – original draft. **Pule David Molebatsi:** writing – original draft. **Lesego Molwelang:** writing – original draft. **Alain Mwamba Mukendi:** conceptualization, writing – original draft, writing – review and editing.

## Funding

The authors have nothing to report.

## Consent

Written informed consent was obtained from the patient for publication of this manuscript and accompanying pictures. A copy of the written consent is available for review by the Editor‐in‐Chief of this journal.

## Conflicts of Interest

The authors declare no conflicts of interest.

## Data Availability

The data that support the findings of this study are available on request from the corresponding author. The data are not publicly available due to privacy or ethical restrictions.
